# Herbal Prescription, DSGOST, Prevents Cold-Induced RhoA Activation and Endothelin-1 Production in Endothelial Cells

**DOI:** 10.1155/2014/549307

**Published:** 2014-04-15

**Authors:** Sung-Gook Cho, Ho Yeon Go, Jeong-Su Park, Ki-Yong Jung, Seung-Ho Sun, You-Kyung Choi, Yun-Kyung Song, Jong-Hyeong Park, Chan-Yong Jun, Seong Gyu Ko

**Affiliations:** ^1^Department of Preventive Medicine, College of Korean Medicine, Kyung Hee University, Seoul 130701, Republic of Korea; ^2^Department of Korean Internal Medicine, College of Korean Medicine, Semyung University, Chungju 380960, Republic of Korea; ^3^Department of Korean Internal Medicine, College of Korean Medicine, Gachon University, Seongnam 461701, Republic of Korea; ^4^Department of Oriental Internal Medicine, College of Korean Medicine, Sangji University, Wonju 220702, Republic of Korea; ^5^Department of Korean Rehabilitation Medicine, College of Korean Medicine, Gachon University, Seongnam 461701, Republic of Korea

## Abstract

Herbal prescription, Danggui-Sayuk-Ga-Osuyu-Saenggang-tang (DSGOST), has long been used to treat Raynaud's phenomenon (RP) in traditional Chinese medicine (TCM). However, a biological mechanism by which DSGOST ameliorates RP is yet deciphered. In this study, we demonstrate that DSGOST inhibits cold-induced activation of RhoA, in both vascular smooth muscle cells (VSMC) and endothelial cells (EC), and blocks endothelin-1-mediated paracrine path for cold response on vessels. While cold induced RhoA activity in both cell types, DSGOST pretreatment prevented cold-induced RhoA activation. DSGOST inhibition of cold-induced RhoA activation further blocked **α**2c-adrenoreceptor translocation to the plasma membrane in VSMC. In addition, DSGOST inhibited endothelin-1-mediated RhoA activation and **α**2c-adrenoreceptor translocation in VSMC. Meanwhile, DSGOST inhibited cold-induced or RhoA-dependent phosphorylation of FAK, SRC, and ERK. Consistently, DSGOST inhibited cold-induced endothelin-1 expression in EC. Therefore, DSGOST prevents cold-induced RhoA in EC and blocks endothelin-1-mediated paracrine path between EC and VSMC. In conclusion, our data suggest that DSGOST is beneficial for treating RP-like syndrome.

## 1. Introduction

Raynaud's phenomenon (RP) is a well-characterized clinical syndrome defined by cold hypersensitivity and recurrent episodes of digital color changes via vasoconstriction of digital arteries to cold and emotional stresses [1]. RP is classified as primary and secondary RP, which is related to its association with other diseases such as systemic sclerosis [1]. Primary RP as an isolated disease is not dependent from vascular structural abnormalities or digital trophic changes, while secondary RP is associated with vascular abnormalities [1]. Nevertheless, the pathogenesis of RP is not fully understood. Cold causes endothelin-1 production from endothelial cells (EC) [2–5]. Endothelin-1 is one of key factors for vasoconstriction, and its expression level increases in RP patients [2, 3, 6, 7]. Endothelin-1 expression is tightly regulated by one of Rho GTPases, RhoA [8]. Therefore, cold stimulates endothelin-1 production from EC through RhoA activation. Endothelin-1 released from EC promotes RhoA activation in vascular smooth muscle cells (VSMC) [9–11]. This endothelin-1-mediated RhoA activation in VSMC results in a translocation of *α*2c-adrenoreceptor from the Golgi to the plasma membrane [12–16]. *α*2c-Adrenoreceptor mainly regulates cold-mediated vasoconstriction [14, 15, 17, 18]. Therefore, *α*2c-adrenoreceptor is proposed to be one of the targets for RP [19, 20].

Traditional Chinese medicine (TCM) has long been applied to the treatment of RP, and recent TCM-based researches have tried to decipher biological mechanisms on TCM theory-based treatment of RP [21–29]. One of the famous herbal prescriptions, Danggui-Sayuk-Ga-Osuyu-Saenggang-Tang (DSGOST; Danggui-Sini-Jia-Wuzhuyu-Shengjian-Tang in Chinese, Tokishigyakukagoshuyushokyoto in Japanese) has historically long been used for treating RP, since it has been thought to be useful for warming the interior on the basis of TCM theories [25–28, 30]. DSGOST is a mixed herbal medicine which consists of Angelica root, Cinnamon bark, Peony root, Akebia stem, Asarum root, Glycyrrhiza, Jujube, Evodia fruit, and Ginger [25–27, 30]. When rats were orally received with DSGOST, peripheral circulation in rat tails was improved [30]. Therefore, DSGOST treatment appears to be effective in RP treatment. However, biological evidence for DSGOST effect on RP is still lacking.

In this study, we investigated DSGOST effect on* in vitro* cold responses on vascular cells that is similar to vascular condition in RP. DSGOST inhibited cold- or endothelin-1-induced Rho activation in EC and VSMC, respectively. Furthermore, DSGOST blocked RhoA-dependent endothelin-1 expression in EC and RhoA-mediated *α*2c-adrenoreceptor translocation to the plasma membrane. Therefore, our* in vitro* data demonstrate that DSGOST is likely to be effective in RP treatment.

## 2. Materials and Methods

### 2.1. Herbal Extracts

Danggui-Sayuk-Ga-Osuyu-Senggang-Tang (DSGOST) was prepared by extracting mixed components as follows: 1 g of Angelica radix (danggui in Korean and Chinese), 1 g of Cinnamomi cortex (geji in Korean, guizhi in Chinese), 1 g of* Paeoniae* root (jakyak in Korean, bai shao in Chinese), 1 g of Akebia root (moktong in Korean, mutong in Chinese), 0.67 g of Asarum (sesin in Korean, xixin in Chinese), 0.67 g of Glycyrrhiza (gamcho in Korean, gancao in Chinese), 1.67 g of* Zizyphus jujuba* (daechu in Korean, dazao in Chinese), 0.67 g of Evodia fruit (osuyu in Korean, wuzhuyu in Chinese), and 1.33 g of Ginger root (saenggang in Korean, shengjian in Chinese). In detail, those components above put in water of 10-fold volume were extracted by heating at 100°C for 2 hours. Extracts were then filtered, concentrated with low pressure, and then dried to make DSGOST powder.

### 2.2. Cell Culture

Human dermal microvascular endothelial cells (HDMEC) and umbilical vein endothelial cells (HUVEC) were obtained from ScienCell Research Laboratories (Carlsbad, CA, USA) and cultured in endothelial cell medium supplemented with 5% fetal bovine serum, 1% endothelial cell growth supplement, and 1% penicillin/streptomycin solution (ScienCell Research Laboratories, Carlsbad, CA, USA). Vascular smooth muscle cells (VSMC) from the human umbilical arteries (HUASMC, ScienCell Research Laboratories, Carlsbad, CA, USA) were cultured in smooth muscle cell medium supplemented with 2% fetal bovine serum, 1% smooth muscle cell growth supplement (ScienCell Research Laboratories, Carlsbad, CA, USA), and 1% penicillin/streptomycin solution.

### 2.3. *In Vitro* Studies

Cells were cultured in different temperatures for 30 minutes to examine cold responses. For endothelin-1-mediated responses, cells were treated with endothelin-1 (Sigma-Aldrich, St. Louis, MO, USA). Cells were transfected with constitutively active or dominantly negative mutant form of RhoA for 48 hours to examine RhoA-dependent mechanisms [31]. The activity of Rho GTPases was determined by GST-pull-down assays as described in previous studies [32, 33]. RhoA was detected using RhoA antibody purchased from Santa Cruz Biotechnology (Santa Cruz, CA, USA). Endothelin-1 production in endothelial cells (EC) was determined using Endothelin-1 Quantikine ELISA kit according to the manufacturer's instruction (R&D systems, Minneapolis, MN, USA). Antibodies for pFAK and pSRC were obtained from Cell Signal (Danvers, MA, USA). Antibodies for Actin and pERK were purchased from SantaCruz Biotechnology (Santa Cruz, CA, USA). An antibody for *α*2c-adrenoreceptor was obtained from Abcam (Cambridge, UK). Plasma membrane protein was isolated using Plasma membrane protein extraction kit (Abcam, Cambridge, UK) according to manufacturer's instruction. *α*1-Na^+^, K^+^-ATPase, was detected as an internal control for plasma protein using anti-*α*1-Na^+^, K^+^-ATPase antibody (SantaCruz Biotechnology, Santa Cruz, CA, USA). Serum response element (SRE) reporter assays were done as described in a previous study [31]. In brief, endothelial cells were transfected with SRE-luc plasmid and then subjected to the luciferase assays. Experiments were done in triplicate and repeated three times independently. Total RNA was isolated with Trizol reagent (Life Technology, Grand Island, NY, USA), and cDNA was synthesized using reverse transcription-polymerase chain reaction (RT-PCR). Real-time PCRs for* Endothelin-1* and glyceraldehyde-3-phosphoate dehydrogenase (*GAPDH*) mRNA level were conducted using primers as follows:* endothelin-1* forward primer: 5′-TGCTCCTGCTCGTCCCTGAT-3′ and reverse primer: 5′-TAACGCTCTCTGGAGGGCTT-3′,* GAPDH* forward primer: 5′-GTGTCGCTGTTGAAGTCAGA-3′, reverse primer: 5′-TGAAGGTCGGAGTCAACGGA-3′ [34, 35]. Relative* endothelin-1* mRNA levels were calculated by ΔΔCt values. Real-time PCRs were conducted in triplicate and repeated three times independently. To detect stress fiber and focal adhesion complex formation, cells were stained with rhodamine-phalloidin (Life Technology, Grand Island, NY, USA).

## 3. Results 

### 3.1. DSGOST Inhibits RhoA Activation and *α*2c-Adrenoreceptor Translocation in Vascular Smooth Muscle Cells

**Figure 1:**
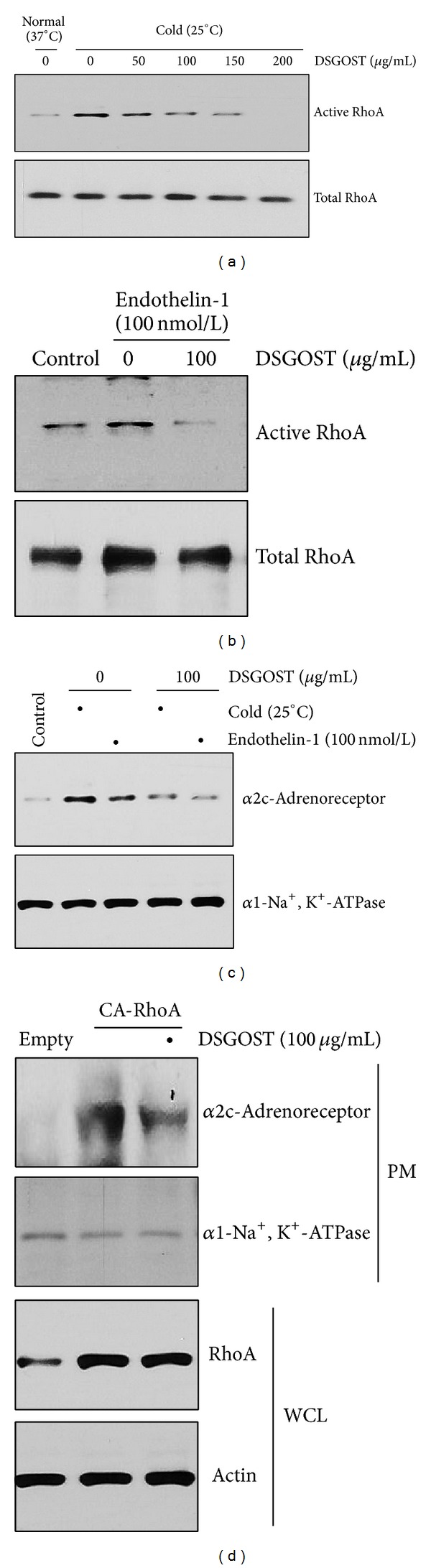
DSGOST inhibition of cold-induced responses in VSMC. (a) DSGOST inhibition of cold-induced RhoA activation in VSMC. Cells were pretreated with different concentrations (50, 100, 150, and 200 *μ*g/mL) of DSGOST for 30 minutes and then exposed to cold temperature (25°C) for another 30 minutes. Active and total RhoA were blotted using anti-RhoA antibody. (b) DSGOST inhibition of endothelin-1-induced RhoA activation in VSMC. Cells were pretreated with 100 *μ*g/mL of DSGOST for 30 minutes and then treated with 100 nmol/L of endothelin-1 for another 30 minutes. (c) Membrane translocation of *α*2c-adrenoreceptor. Cells were pretreated with DSGOST (100 *μ*g/mL) for 30 minutes and then either exposed to cold (25°C) or treated with endothelin-1 (100 nmol/L) for 30 minutes. Plasma membrane protein was isolated and then *α*2c-adrenoreceptor was detected using the appropriate antibody. (d) RhoA-mediated *α*2c-adrenoreceptor translocation to the membrane. VSMC were transfected with CA-RhoA and then treated with DSGOST (100 *μ*g/mL) for 30 minutes. *α*2c-Adrenoreceptor was detected in the plasma membrane protein pool. *α*1-Na^+^, K^+^-ATPase, was detected as the internal control for plasma protein.

As cold-induced RhoA activation in VSMC has been revealed [1, 15, 36], we first examined DSGOST effect on cold-induced RhoA activation in VSMC. When VSMC were pretreated with different concentrations (50, 100, 150, and 200 *μ*g/mL) of DSGOST for 30 minutes and then cultured in cold temperature (25°C) for another 30 minutes, DSGOST blocked cold-induced RhoA activation (Figure 1(a)). Endothelin-1 produced from EC has been known to result in a contraction of VSMC via RhoA activation [1, 7, 9, 11]. Therefore, we examined DSGOST effect on endothelin-1-induced RhoA activation in VSMC. When VSMC were pretreated with 100 *μ*g/mL of DSGOST for 30 minutes and then treated with endothelin-1 (100 nmol/L) for another 30 minutes, DSGOST inhibited endothelin-1-induced RhoA activation (Figure 1(b)). Therefore, DSGOST inhibits RhoA activation induced by both cold and endothelin-1.

Cold-induced or endothelin-1-mediated RhoA activation has been known to result in *α*2c-adrenoreceptor translocation to the plasma membrane in VSMC [1, 7, 12–14, 18, 19, 36]. When VSMC were pretreated with DSGOST (100 *μ*g/mL) for 30 minutes and then exposed to either cold (25°C) or treated with endothelin-1 (100 nmol/L) for 30 minutes, DSGOST blocked cold- or endothelin-1-induced *α*2c-adrenoreceptor localization at the plasma membrane in VSMC (Figure 1(c)).

In addition, when VSMC were overexpressed with constitutively active form of RhoA (CA-RhoA), DSGOST failed to inhibit RhoA-mediated *α*2c-adrenoreceptor translocation to the plasma membrane in VSMC (Figure 1(d)). Therefore, our data indicate that DSGOST inhibition of RhoA activity may be crucial for ameliorating cold effect on VSMC.

### 3.2. DSGOST Inhibits Cold-Induced RhoA Activation in Endothelial Cells

**Figure 2:**
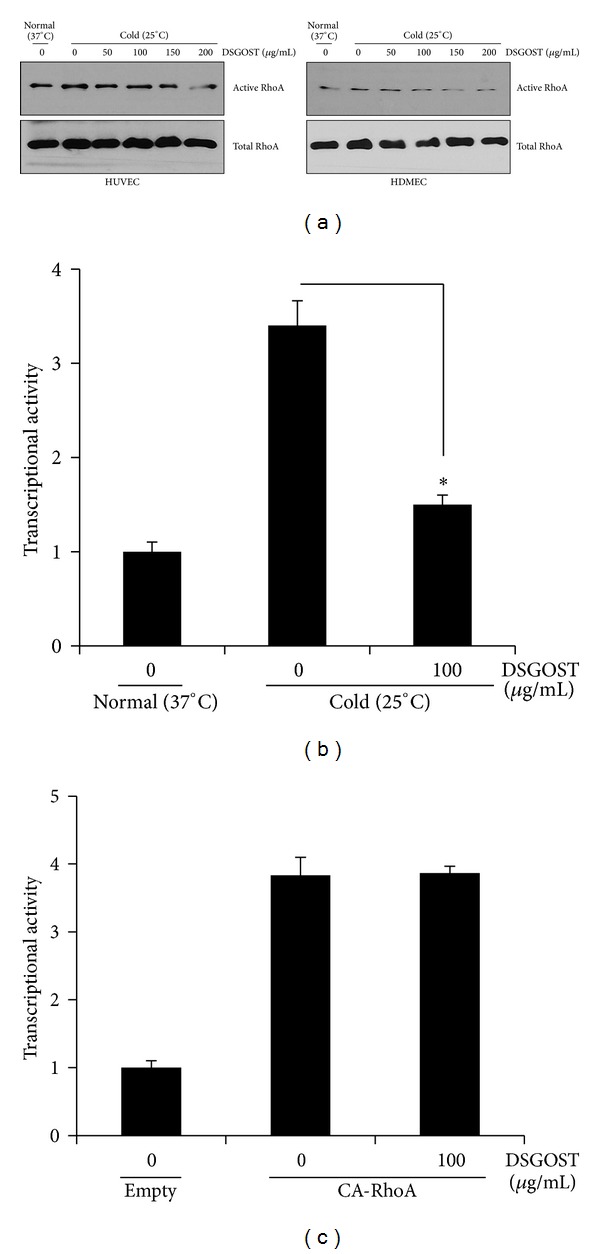
DSGOST inhibition of cold-induced RhoA activation in endothelial cells. (a) HUVEC or HDMEC were pretreated with DSGOST at different concentrations (50, 100, 150, and 200 *μ*g/mL) for 30 minutes, exposed to 25°C for another 30 minutes, and then subjected to GST pull-down assays for RhoA activity. (b) Cells were transfected with SRE-luc construct for 48 hours, pretreated with DSGOST (100 *μ*g/mL) for 30 minutes, and then exposed to cold (25°C) for another 90 minutes,  *P < 0.05. (c) Cells were transfected with CA-RhoA and SRE-luc constructs for 48 hours, treated with DSGOST (100 *μ*g/mL) for 2 hours, and then subjected to the reporter assays. Experiments were performed in triplicate and repeated three times independently. Data represent mean ± SEM.

To examine if cold exposure affects RhoA activity in EC, either HDMEC or HUVEC were cultured in 25°C or 37°C for 30 minutes and then subjected to GST-pull-down assays for RhoA activity. Cold exposure increased RhoA activity (Figure 2(a)), indicating that cold induces RhoA activation even in the endothelial cell. When HDMEC or HUVEC were pretreated with different concentrations (50, 100, 150, and 200 *μ*g/mL) of DSGOST for 30 minutes and then cultured in 25°C for another 30 minutes, DSGOST blocked cold-induced RhoA activation in a dose-dependent manner (Figure 2(a)). Thus, our data indicate that DSGOST inhibits cold-induced RhoA activation in EC.

The well-known readout for RhoA activity is an activity of serum response element (SRE) [31]. Therefore, we further examined if DSGOST affects SRE reporter activity. In SRE reporter assays, while cold (25°C) exposure for 90 minutes increased SRE reporter activity by approximately fourfold, DSGOST pretreatment (100 *μ*g/mL) for 30 minutes repressed cold-induced SRE reporter activity in HDMEC (Figure 2(b)). In addition, when HDMEC were overexpressed with CA-RhoA for 48 hours and then treated with DSGOST (100 *μ*g/mL) for 2 hours, DSGOST failed to inhibit RhoA-induced SRE reporter activity (Figure 2(c)).

### 3.3. DSGOST Inhibition of RhoA-Dependent FAK Phosphorylation

**Figure 3:**
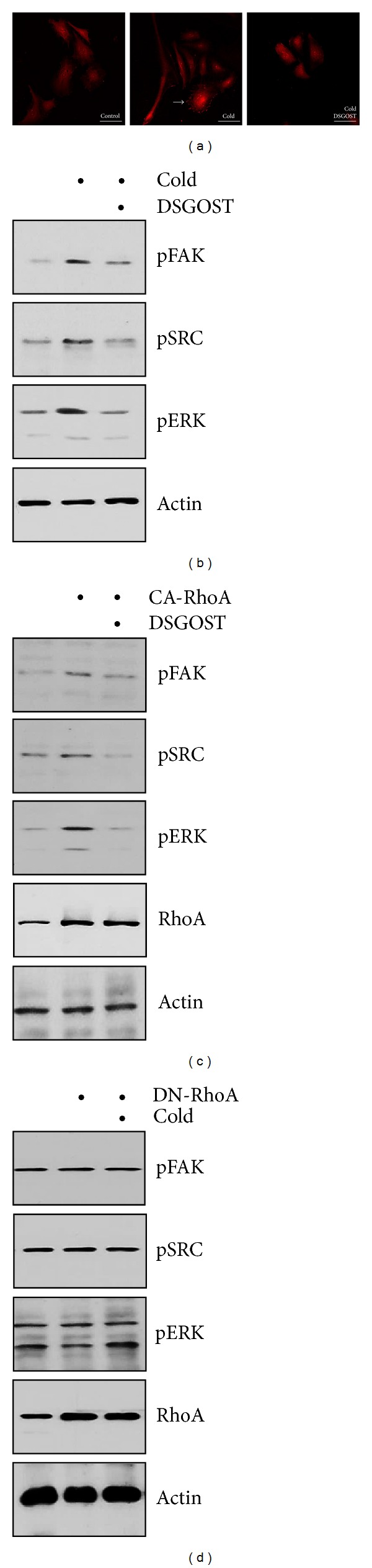
DSGOST inhibition of RhoA-mediated FAK phosphorylation. (a) HDMEC were pretreated with DSGOST (100 *μ*g/mL) for 30 minutes and then exposed to cold (25°C) for another 30 minutes. F-actin was detected using rhodamine-phalloidin. The arrow indicates the representative of focal adhesion complex. Scale bars indicate 500 *μ*m. (b) HDMEC were pretreated with DSGOST (100 *μ*g/mL) for 30 minutes and then exposed to cold (25°C) for another 30 minutes. (c) HDMEC were transfected with CA-RhoA for 48 hours and then treated with DSGOST (100 *μ*g/mL) for 30 minutes. (d) HDMEC were overexpressed with DN-RhoA and then exposed to cold (25°C) for another 30 minutes. Phosphorylated forms of FAK, SRC, and ERK were detected with appropriate antibodies. Actin was detected as the internal control.

RhoA activation leads to FAK phosphorylation, resulting in the formation of stress fiber and focal adhesion complex. While cold (25°C) increased the formation of stress fiber and focal adhesion complex in EC, DSGOST (100 *μ*g/mL) inhibited the formation of stress fiber and focal adhesion complex (Figure 3(a)). Consistently, DSGOST inhibited cold-induced FAK phosphorylation (Figure 3(b)). Therefore, our data show that DSGOST inhibits cold-induced FAK phosphorylation and formation of focal adhesion complex and stress fiber. Next, we examined if cold induces phosphorylation of SRC and ERK, since FAK phosphorylation results in phosphorylation of SRC and ERK. While cold exposure caused phosphorylation of SRC and ERK in HDMEC, DSGOST inhibited cold-induced phosphorylation of SRC and ERK (Figure 3(b)).

We further examined whether cold causes phosphorylation of FAK, SRC and ERK via RhoA. Whereas RhoA activation increased phosphorylation of FAK, SRC, and ERK in HDMEC overexpressing CA-RhoA, DSGOST failed to inhibit CA-RhoA-mediated phosphorylation of FAK, SRC, and ERK (Figure 3(c)). Next, when cells were overexpressed with dominant negative mutant form of RhoA (DN-RhoA), cold did not induce phosphorylation of FAK, SRC, and ERK (Figure 3(d)). Therefore, DSGOST inhibition of cold-induced RhoA activation is a key for phosphorylation of FAK, SRC, and ERK.

### 3.4. DSGOST Inhibits RhoA-Dependent* Endothelin-1* Expression

**Figure 4:**
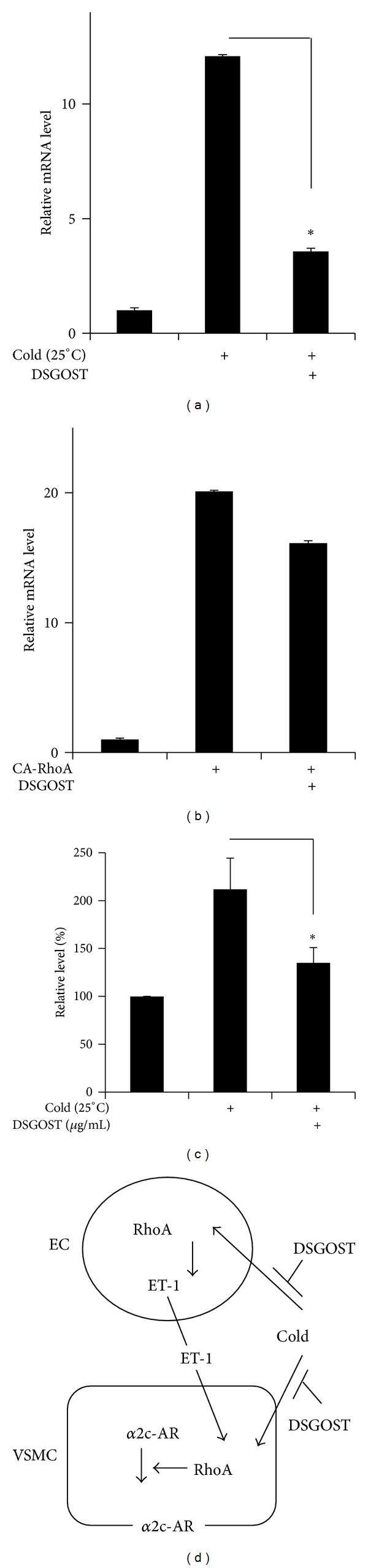
DSGOST inhibition of cold-induced endothelin-1 expression. (a) HDMEC were pretreated with DSGOST (100 *μ*g/mL) for 30 minutes and then exposed to cold (25°C) for another 4 hours. Relative* endothelin-1 *mRNA expression level was examined using real-time PCR, *P < 0.05. (b) HDMEC were transfected with CA-RhoA for 48 hours and then treated with DSGOST (100 *μ*g/mL) for 4 hours. Relative* endothelin-1* mRNA level was detected using real-time PCR. (c) Endothelin-1 level from HDMEC-cultured medium was measured by ELISA. Experiments were performed in triplicate. Bars indicate the mean ± SD,  *P < 0.05. (d) Schematic cartoon. DSGOST inhibits cold-induced RhoA activation, resulting in repression of endothelin-1 (ET-1) production from EC. Subsequently, ET-1-induced RhoA activation and *α*2c-adrenoreceptor (*α*2c-AR) translocation to the membrane are blocked in VSMC. In addition, DSGOST may directly inhibit cold-induced responses in VSMC.

It has been known that cold causes endothelil-1 upregulation in EC [4, 5, 8, 34]. Therefore, we further examined if DSGOST affects expression level of endothelin-1 in HDMEC. Whereas cold (25°C) exposure for 4 hours increased* endothelin-1* mRNA expression in HDMEC by approximately 10-fold, DSGOST (100 *μ*g/mL) pretreatment for 30 minutes repressed cold-induced* endothelin-1* mRNA expression (Figure 4(a)). However, DSGOST failed to inhibit RhoA-induced* endothelin-1* mRNA expression, when HDMEC were transfected with CA-RhoA for 48 hours and then treated with DSGOST (100 *μ*g/mL) for another 4 hours (Figure 4(b)). Accordingly, DSGOST blocked cold-induced endothelin-1 production in HDMEC, when endothelin-1 level from the medium was measured (Figure 4(c)). Therefore, DSGOST inhibition of RhoA activation results in reduction of endothelin-1 expression in EC.

## 4. Discussion

DSGOST has long been used for treating Raynaud's phenomenon in TCM [25–28]. However, its biological mechanism has not been clearly reported. In this study, we found that DSGOST inhibits cold-induced responses in both EC and VSMC. Moreover, our study revealed that RhoA is a key player for cold response on both EC and VSMC.

Cold-induced intra- and intercellular signaling paths in EC and VSMC have been deciphered, while whole mechanisms are yet clearly defined [1, 6, 12, 15, 16, 19, 36]. While researches for cold-induced vasoconstriction have revealed a crucial role of RhoA in VSMC, our data suggest that RhoA activity in both EC and VSMC would be one of readouts for cold-induced vascular cellular responses [8, 11, 12, 15, 16]. In addition, although we still need to define what chemical components in DSGOST affect RhoA activity, one of biological roles of DSGOST is likely to inhibit RhoA activity independently of either vascular cell types or environmental cues since DSGOST inhibited cold-induced RhoA activation in both EC and VSMC. Thus, DSGOST in TCM would be beneficial for RP treatment on the basis of our* in vitro* study, while its biological mechanism remains to be clearly revealed [25–27, 29]. Furthermore, DSGOST inhibition of RhoA shown in our data would be a hint for DSGOST application to other diseases relating to RhoA deregulation [37–39]. In case of chronic obstructive pulmonary disease (COPD), pulmonary endothelial dysfunction is tightly linked to RhoA deregulation [38]. In chronic kidney disease (CKD), RhoA deregulation is likely to be related to podocyte dysfunction [37]. Likewise, RhoA deregulation appears to be involved in a progression of diabetic nephropathy [39]. All those cases mentioned above address a crucial role of RhoA in EC. Therefore, it is worth investigating if DSGOST effect on RhoA in EC would be beneficial for treating particular diseases related to EC dysfunction.

Endothelin-1 production in EC is one of keys for cold-induced vasoconstriction [6, 12, 15]. Our data showed that cold exposure and endothelin-1 treatment activate RhoA in VSMC, resulting in *α*2c-adrenoreceptor translocation to the plasma membrane. Moreover, DSGOST inhibition of cold-induced RhoA activation reduced endothelin-1 expression in EC, which indicates that DSGOST blocks endothelin-1-mediated paracrine path for vasoconstriction (Figure 4(d)). Therefore, DSGOST inhibition of endothelin-1 production seems to prolong its anti-vasoconstrictive effect [6, 7, 40].

As mentioned, whereas DSGOST has long been used in TCM to treat RP, its therapeutic mechanisms have not been studied even in the* in vitro* experimental conditions [25, 26]. While we need more evidence to convince DSGOST effect on RP, this study first demonstrates DSGOST effect on cold-exposed vascular cells. Therefore, our further studies will try to prove how DSGOST ameliorates RP* in vivo* and to decipher what components in DSGOST are effective in both* in vitro* and* in vivo* experimental systems, which will help to answer a historical reason of DSGOST prescription for RP treatment.

## 5. Conclusion

While DSGOST has long been used in TCM to treat diseases such as RP, its effect has not been proven in experimental systems. This study first reveals DSGOST effect on RhoA in vessel cells. Furthermore, our data show DSGOST inhibition of endothelin-1-mediated paracrine path between EC and VSMC. In conclusion, DSGOST is useful for treating malfunction of cold-induced vessels.
